# The 3-Base Periodicity and Codon Usage of Coding Sequences Are Correlated with Gene Expression at the Level of Transcription Elongation

**DOI:** 10.1371/journal.pone.0021590

**Published:** 2011-06-28

**Authors:** Edoardo Trotta

**Affiliations:** Institute of Translational Pharmacology, Consiglio Nazionale delle Ricerche, Roma, Italy; University of Edinburgh, United Kingdom

## Abstract

**Background:**

Gene transcription is regulated by DNA transcriptional regulatory elements, promoters and enhancers that are located outside the coding regions. Here, we examine the characteristic 3-base periodicity of the coding sequences and analyse its correlation with the genome-wide transcriptional profile of yeast.

**Principal Findings:**

The analysis of coding sequences by a new class of indices proposed here identified two different sources of 3-base periodicity: the codon frequency and the codon sequence. In exponentially growing yeast cells, the codon-frequency component of periodicity accounts for 71.9% of the variability of the cellular mRNA by a strong association with the density of elongating mRNA polymerase II complexes. The mRNA abundance explains most of the correlation between the codon-frequency component of periodicity and protein levels. Furthermore, pyrimidine-ending codons of the four-fold degenerate small amino acids alanine, glycine and valine are associated with genes with double the transcription rate of those associated with purine-ending codons.

**Conclusions:**

We demonstrate that the 3-base periodicity of coding sequences is higher than expected by the codon usage frequency (CUF) and that its components, associated with codon bias and amino acid composition, are correlated with gene expression, principally at the level of transcription elongation. This indicates a role of codon sequences in maximising the transcription efficiency in exponentially growing yeast cells. Moreover, the results contrast with the common Darwinian explanation that attributes the codon bias to translational selection by an adjustment of synonymous codon frequencies to the most abundant isoaccepting tRNA. Here, we show that selection on codon bias likely acts at both the transcriptional and translational level and that codon usage and the relative abundance of tRNA could drive each other in order to synergistically optimize the efficiency of gene expression.

## Introduction

Gene transcription by the RNA polymerase II machinery is regulated by interactions between transcription factors and specific DNA sites. Transcription factors act before the RNA-elongation stage by binding to the promoter and enhancer DNA regions located outside the coding sequences. Recently, it has emerged that transcription is also regulated at the level of elongation by the activity of RNA polymerase II elongation factors [1], [2]. However, while the identity and role of elongation factors are becoming progressively clarified, whether and eventually how the coding regions of transcribed DNA participate in the transcriptional regulation is unknown.

Some recent experimental evidence suggests that regulation at the level of transcription elongation in yeast is associated with coding regions. For example, the enrichment of RNA polymerase II, relative to its transcription rate, is detected in intron-less ribosomal protein genes and, at least for RPS3 and RPL25, inactive polymerases accumulate along the length of the gene with some bias toward their 5′ moiety [2]. Moreover, the RTF1 and SPT5 elongation factors and the CHD1 chromatin remodelling factor associate with the coding regions of actively transcribed chromatin, which also suggests a regulatory role of chromatin remodelling in transcription elongation [3]. These findings suggest the attractive hypothesis that coding sequences play a regulatory role at the level of transcription elongation.

Differently from the well-studied regulatory elements of promoters, the sequence of the coding DNA is constrained by the amino acid sequence of the corresponding encoded protein. However, because most of the 20 amino acids are encoded by more than one codon (synonymous codons), changes to the coding sequences can occur without altering the amino acid sequence. An exchange between non-synonymous codons is also tolerated if the resulting protein maintains proper functionality. Therefore, any codon adjustment to maximize transcription efficiency should produce changes to the DNA primary structure that are correlated with transcription levels.

A characteristic primary structure of coding regions among all known organisms that is linked to codon composition is the 3-base periodicity [4], [5]. This structural property has been exploited in bioinformatics tools for predicting genomic coding sequences [6], for finding potential shifts of reading frame [7] and for the analysis of gene evolution [8]. However, its origin has not been fully clarified, and two principal classes of hypotheses have been reported: those considering the codon or amino acid frequencies as the only cause [9], [10] and those emphasizing the role of the amino acid sequence [11]. This difference is not negligible, considering that codon bias (synonymous codons are not used at the same frequency) is correlated with cellular tRNA abundance and gene expression levels [12]. The commonly accepted theory known as the mutation-selection-drift balance model of synonymous codon usage [13], [14] assumes that the high frequency of optimal synonymous codons is maintained by selection, while neutral mutational pressure and genetic drift allow the minor codons to maintain their low frequency. The major cause for the selection of codon bias, although not fully validated, is generally attributed to translational forces: codons with more abundant cognate tRNA are translated more efficiently and correctly because they reduce ribosome pausing during elongation and decrease the probability of incorporating incorrect amino acids [15], [16], [17].

A role for codon order in gene expression has also been suggested. In *E. coli*, codon-pair usage is different between highly and poorly expressed genes [18] and is correlated with translational elongation rate in vivo [19]. Moreover, in *S. cerevisiae* the synonymous codons associated to a common tRNA tend to be reused for successive coding of the same amino acid especially in rapidly induced genes and accelerate translation [20].

This work proposes a new approach and new indices for detecting and quantifying 3-base periodicity with the aim of determining whether there is a correlation between the primary structure of coding sequences and gene expression. The results reported here show new findings on the nature of the 3-base periodicity of coding sequences and on the association between gene expression and 3-base periodicity.

## Results

### Model validation and the 3-base periodicity of artificial coding sequences

The analysis of the 3-base periodicity was performed on coding sequences of bacterial genomes with different GC content, including those of *Mycobacterium tuberculosis* (G+C = 65.6%), *Escherichia coli* (G+C = 50.8%), *Bacillus subtilis* (G+C = 43.5%), and the eukaryotic genome of *Saccharomyces cerevisiae*. The periodicity was studied by analysing the frequency distribution of the discrete variable D_XY_, which measures the distance at which nucleotide Y occurs for the first time after nucleotide X. To elucidate the role of the codon composition and the codon sequence in the D_XY_ frequency distribution of CDSs, two different pseudo-random sequences were generated by calculation and simulation procedures: random sequences with specified nucleotide frequencies (RandNuc) and random sequences with specified codon frequencies (RandCod). In addition, randomized native sequences were generated by shuffling codons within the coding sequences (ShufCod).

**Figure 1:**
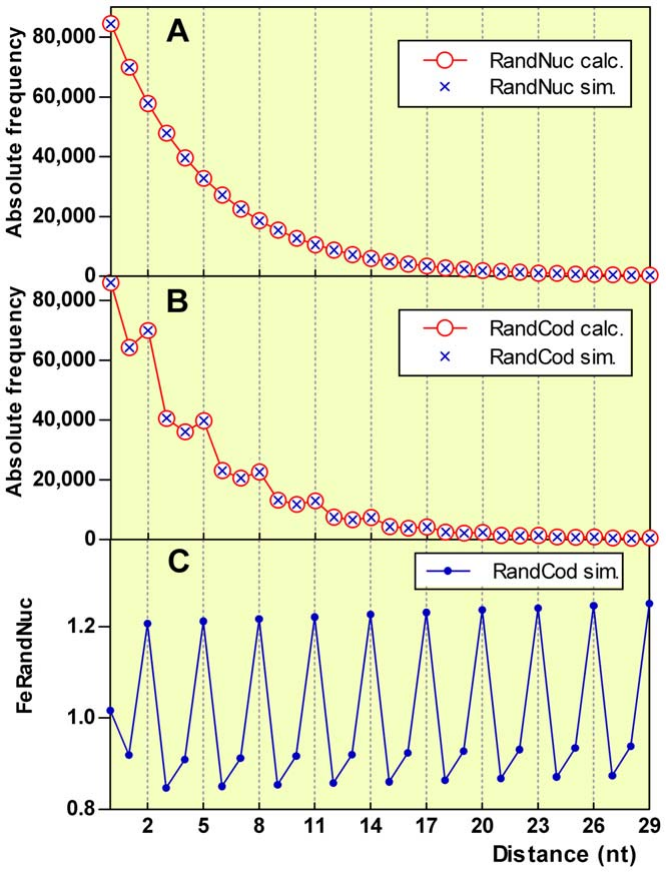
Artificial random sequences based on nucleotide and codon frequencies of *M. tuberculosis*. Absolute frequency distribution of D_TT_ estimated by 5000 simulations (blue x) or calculated (red o) for RandNuc (panel **A**) and RandCod (panel **B**) sequences. Panel **C** shows the frequency distribution of the simulated RandCod D_TT_ in units of RandNuc D_TT_ (FeRandNuc).

The frequency distributions of D_TT_ in the RandNuc and RandCod sequences based on nucleotide and codon frequencies of *M. tuberculosis* are illustrated in Figure 1. As shown, the expected D_TT_ frequencies calculated by equations (2) and (3) overlap with the corresponding average values of 5000 simulated sequences, thus validating the robustness of the models. Also, the RandCod sequences display a 3-base periodicity that is absent from the geometric distribution of the RandNuc sequences. The 3-base periodicity is more apparent in Figure 1C, in which the D_TT_ frequencies are plotted in units of expected frequencies calculated for the RandNuc model. We define this ratio as the frequency equivalent RandNuc or FeRandNuc.

**Figure 2:**
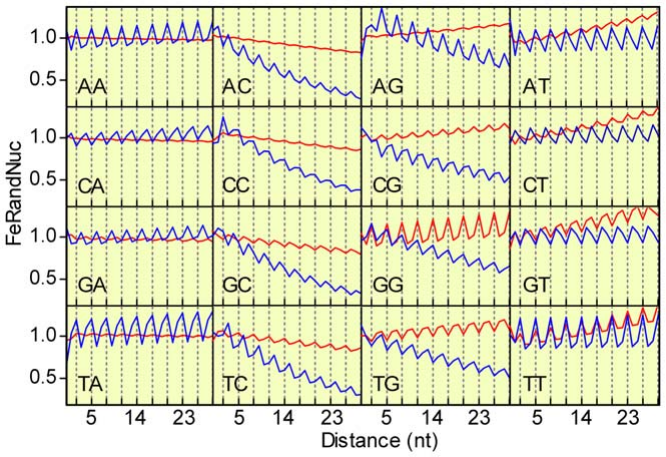
RandCod artificial sequences based on the native codon frequencies of *M. tuberculosis*. Expected FeRandNuc of D_XY_ frequency distributions for the 16 dinucleotides before (blue line) and after (red line) the equalization of synonymous codons.

Figure 2 (blue lines) shows all of the D_XY_ frequency distributions corresponding to the RandCod sequences based on the codon usage of *M. tuberculosis*. As shown, all couples of bases exhibited equal periods corresponding to three nucleotides but with varying amplitude and phase shift. A period length of three was also detected using the codon composition of the other genomes analysed in this work but not using a codon length different from 3 nucleotides. From Fig.2, the FeRandNuc of NC and NG shows a different variation along distance than NA and NT, which was not observed in all the genomes examined here. Moreover, from Figure 2 (blue lines), it is also apparent that the first two values of the FeRandNuc distributions are generally out of phase and are of anomalous intensities.

To investigate the effect of homogeneity of codon composition on the D_XY_ frequency distribution, simulated RandCod sequences were generated using equal frequencies for all 64 codons. The resulting distribution shows the absence of any periodicity (Figure S1). When stop codons (TAA, TAG e TGA) were excluded from the simulated sequences, to mimic their absence from real CDSs, and equal frequencies were set for the remaining 61 codons, some dinucleotides such as TT exhibited a clear periodicity (Figure S1), showing that coding sequences should always display a 3-base periodicity.

**Figure 3:**
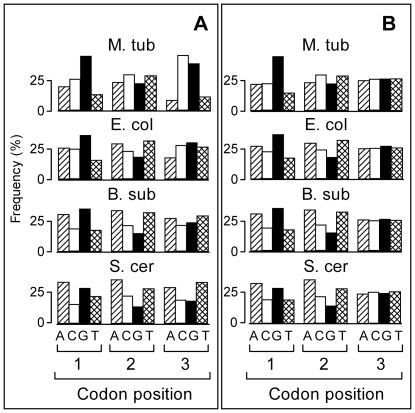
Base frequencies at codon position. Base frequency (%) at each codon position for coding sequences with native (panel **A**) and equalized (panel **B**) frequencies of synonymous codons for different organisms. The first and last codons of each coding sequence were excluded from the count of base frequencies.

We also evaluated the role of codon bias in the 3-base periodicity of D_XY_. Codon bias denotes the general tendency of organisms to unequally use of codons encoding the same amino acid (synonymous codons). We generated RandCod sequences by using the frequencies of synonymous codons equal to the average value of the corresponding family. Figure 3 shows the relative proportions of nucleotides in the three codon positions before (panel A) and after (panel B) frequency equalizations of synonymous codons. As shown, levelling synonymous codons slightly affected the relative nucleotide frequencies at the first- and second-codon positions of the reading frame, while appreciable changes were observed at the third position in all of the analysed organisms. The D_XY_ frequency distributions before (blue lines) and after (red line) the equalization of synonymous codons are illustrated in Figure 2. From the figure, it is apparent that the levelling of synonymous codons produces an increase in the GG periodicity amplitude and varying levels of reduction in all of the other dinucleotides. This result should reflect the effect of equalization in the relative distribution of nucleotides in the three codon positions (Figure 3): A, C and T are more homogeneously distributed, while the relative frequency of G in the first position is increased. Therefore, the expected contribution of codon bias in the coding regions of *M. tuberculosis* is to reduce the GG periodicity and to increase all of the other dinucleotide periodicities. In general, we found that levelling synonymous codons produces different effects on the 3-base periodicity among distinct organisms. For instance, the GG periodicity, which is largely decreased by codon bias in *M. tuberculosis*, is increased in *S. cerevisiae* and *B. subtilis* (data not shown). These different effects make the expected association between the periodicity of individual dinucleotides and codon bias an element of variability among different organisms.

**Figure 4:**
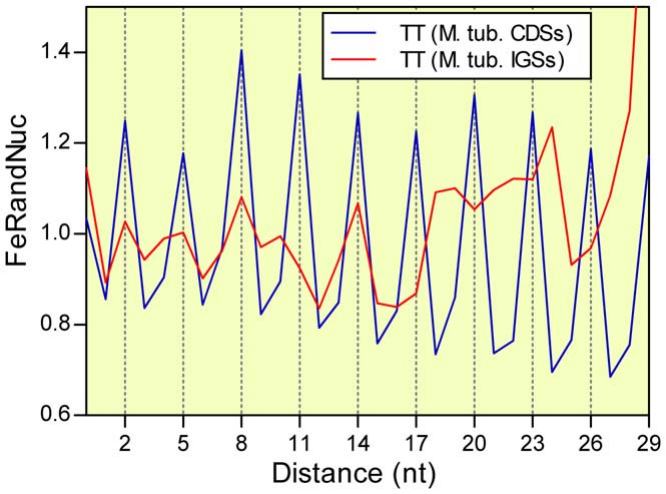
D_TT_ frequency distributions in CDSs and IGs of *M. tuberculosis*. Comparison between D_TT_ frequency distributions in protein coding sequences (CDSs, blue line) and intergenic sequences (IGSs, red line) of *M. tuberculosis*.

The variable D_XY_ was also tested for comparing the 3-base periodicity of CDSs and intergenic regions (IGs) of *M. tuberculosis*. The results for all of the 16 couples of nucleotides are consistent with the specificity of the 3-base periodicity for coding sequences. Figure 4 illustrates the D_TT_ frequency distributions of CDSs and IGs belonging to the *M. tuberculosis* genome.

### The origin and nature of 3-base periodicity of native coding sequences

To investigate the origin and nature of 3-base periodicity in coding DNA, we analysed the significant discrepancies between the D_XY_ frequency distributions of native CDSs and different random sequences. First, we compared the D_XY_ frequency distributions of CDSs with RandCod sequences generated at the same codon composition. The results showed that the 3-base periodicity of each species examined here displays, as a fingerprint, its own characteristic combination of phase shift, period and amplitude parameters related to the 16 dinucleotides, which were mostly reproduced by the corresponding RandCod sequences. The major differences between real and simulated sequences were detected in the amplitude of the 3-base periodicity of some dinucleotides. For instance, in *M. tuberculosis*, the amplitudes of the periodicity of D_AA_, D_CT_, D_GT_ and particularly D_TT_ frequencies are sensitively higher than that expected for the related RandCod sequences. In the particular case of D_TT_, the estimated amplitudes obtained by a sinusoidal curve fitting of real and simulated data (see Figure S2) were equal to 0.307±0.013 and 0.228±0.001, respectively.

**Figure 5:**
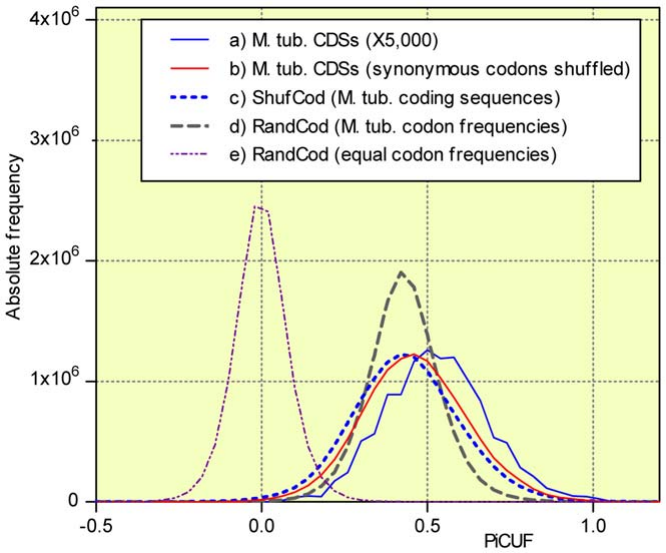
PiCUF in CDSs of *M. tuberculosis* and artificial sequences. Frequency distribution diagrams of PiCUF in CDSs of *M. tuberculosis* (a) and artificial sequences: *M. tuberculosis* CDSs with synonymous codons shuffled (b), *M. tuberculosis* CDSs with codons shuffled (ShufCod) (c), RandCod based on *M. tuberculosis* codon frequencies (d), RandCod at equal codon frequencies (e). Absolute frequencies of real CDSs were normalized against frequencies of simulated sequences by multiplying by 5000.

**Figure 6:**
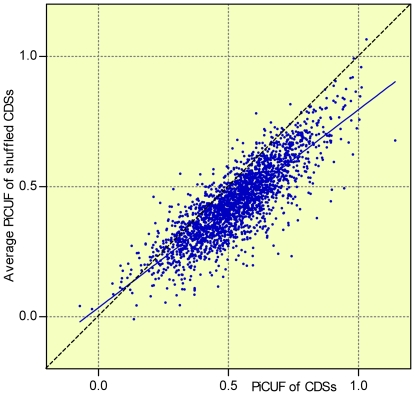
PiCUF of *M. tuberculosis* CDSs versus PiCUF of shuffled CDSs. Scatter plot of the PiCUF of *M. tuberculosis* CDSs versus the average of 5000 PiCUF values obtained by shuffling their codons. The solid line represents the linear regression line and the broken line corresponds to equal values on both axes.

To evaluate the statistical significance of the differences between the CDS and the RandCod periodicities, we introduced a new periodicity index termed PiCUF. This index is a measure of the component of the D_XY_ periodicity in phase with that expected from RandCod sequences based on codon usage frequency (CUF) (see Materials and Methods). The PiCUF was calculated for all CDSs and simulated RandCod, RandNuc and ShufCod sequences. The frequency distribution diagrams of PiCUF values related to *M. tuberculosis* are illustrated in Figure 5. Three main observations emerge from the figure. First, consistent with the above results, the mean PiCUF value of RandCod simulated sequences generated with equal codon frequencies is nearly 0 (mean = −0.0006 and s.d. = 0.083). Second, the PiCUF frequency distributions of codon-shuffled CDSs (ShufCod) and simulated RandCod sequences (codon frequencies equal to CUF) shift to positive values and show similar means (0.430 and 0.436, respectively) but different standard deviations (0.111 and 0.163, respectively) attributable to the non-homogeneous codon frequency distribution among ShufCod sequences. Third and most importantly, the distributions of native CDSs and their shuffled sequences (ShufCod) have similar standard deviations (0.156 and 0.163, respectively) but the mean PiCUF value of the native sequences is significantly higher (means = 0.527 and 0.436, p<0.0001). This is also evident from the scatter plot presented in Figure 6 in which the PiCUF of each CDS is plotted versus the average of 5000 PiCUF values obtained by the shuffling of codons. This unexpected result was obtained for all genomes examined in this work, indicating that the CUF is not fully responsible for the 3-base periodicity detected in real CDSs, but a contribution of the amino acid sequence and/or the arrangement of synonymous codons should be considered. To assess these contributions with respect to CDS periodicity, a random permutation of only synonymous codons was performed within each coding sequence. Such a restricted shuffling leaves the amino acid sequence and codon frequencies unchanged. The resulting PiCUF frequency distribution in the case of *M. tuberculosis* (mean = 0.463, s.d. = 0.160) is illustrated in Figure 5 (line b). As shown, the permutation of synonymous codons produces a strong shift to low PiCUF values of native CDS distribution (line a, mean = 0.527). Moreover, the resulting mean PiCUF value (0.463) is slightly, but significantly (p<0.0001), higher than that of ShufCod (line c, mean = 0.436). This means that about 82.7% of the mean value of PiCUF of native CDSs was expected from codon frequency, 12.1% was due to the non-random arrangement of synonymous codons and the remaining 5.2% was due to the amino acid sequence (Table S1). In order to control if the decrease of PiCUF caused by the shuffling of synonymous codons is associated with mRNA levels, we analyzed separately highly-, moderately- and lowly-expressed mRNA of *S. cerevisiae* of our dataset (408 genes, Dataset S1). An almost invariable decrease of PiCUF index was observed among the highly- (8%), moderately- (7%) and lowly-expressed (10%) mRNAs.

### Correlation of PiCUF with mRNA and cellular protein levels in yeast

As reported above, two sources of 3-base periodicity in CDSs can be distinguished: codon frequency and codon sequence (which is the combination of two components: the amino acid sequence and the positioning of synonymous codons). In *S. cerevisiae*, codon frequency accounted for 85.2% of the PiCUF of real CDSs, and codon sequence accounted for the remaining 14.8% (Table S1, Figure S3). To quantify the relationship of these two components of 3-base periodicity with gene expression, we performed a correlation analysis between PiCUF values and the *S. cerevisiae* gene expression levels reported in the dataset of Lu et al. (2007) [21], [22], [23], [24], [25], [26]. Table 1 shows the Spearman's rank correlation coefficient (R_s_) and the coefficient of determination (R^2^) between the PiCUF index and the codon adaptation index (CAI) [27], protein levels, mRNA levels and protein/mRNA ratio [25] (the corresponding scatter plots are shown in Figure S4). The component of PiCUF due to the codon sequence, calculated as the difference between the PiCUF of native CDSs and the average value corresponding to a 5000 shuffling of its codons, was very weakly correlated with the CAI (R_s_ = 0.047 and R^2^ = 0.011), protein level (R_s_ = 0.054 and R^2^ = 0.006), mRNA level (R_s_ = 0.042 and R^2^ = 0.009) and protein/mRNA ratio (R_s_ = 0.024 and R^2^ = 0.000). However, the data show a surprisingly strong positive correlation between the codon frequency component of PiCUF and the mRNA (R_s_ = 0.779 and R^2^ = 0.558) and protein levels (R_s_ = 0.735 and R^2^ = 0.511). The common relationship of protein and mRNA levels with the frequency component of PiCUF was estimated by calculating the relative partial correlation coefficients. The strong ordinary zero-order Pearson correlation coefficient (R_p_) between the codon frequency component of PiCUF and the protein level (R_p_ = 0.715, p<0.0001) was considerably reduced when the mRNA level was controlled for (partial correlation: R_p_ = 0.227, p<0.0001). In terms of variance, R^2^ was reduced from 0.511 to 0.051, indicating that of the 51.1% of variability in the protein amount accounted for by PiCUF, most (90%) was shared with mRNA level. Consistent with the partial correlation results, the PiCUF frequency component exhibited a moderately weak positive correlation with the protein/mRNA ratio (R_s_ = 0.282, R^2^ = 0.072) (Figure S4 and Table 1). In addition, the frequency component of PiCUF displayed a strong and positive correlation with the codon index CAI (R_s_ = 0.858, R^2^ = 0.660), which measures the similarity of the codon composition of a gene with the codon usage of highly expressed genes [27].

**Table 1:** Correlation coefficients between periodicity indexes and gene expression levels of *S. cerevisiae.*

	CAI^d^		mRNA^d^		Protein^d^		Protein/mRNA^d^	
	R_s_	R^2^	R_s_	R^2^	R_s_	R^2^	R_s_	R^2^
PiCUF	0.810 a	0.602 a	0.733 a	0.507 a	0.701 a	0.459 a	0.279 a	0.061 a
PiCUF freq.	0.858 a	0.660 a	0.779 a	0.558 a	0.735 a	0.511 a	0.282 a	0.072 a
PiCUF seq.	0.047^c^	0.011 b	0.042 c	0.009 c	0.054 c	0.006 c	0.024 c	0.000 c
Pi^d^	0.908 a	0.812 a	0.854 a	0.719 a	0.803 a	0.642 a	0.287 a	0.081 a

Spearman's rank correlation coefficient (R_s_) and coefficient of determination (R^2^) between periodicity indexes and CAI, mRNA level, protein level and protein/mRNA ratio in CDSs of *S. cerevisiae* (N = 408). PiCUF freq. and PiCUF seq. indicate frequency and sequence components of PiCUF, respectively.

a *P*<0.0001.

b *P*<0.05.

c *P*>0.05.

d Log-transformed data.

### Correlation of Pi with mRNA and cellular protein levels in yeast

**Figure 7:**
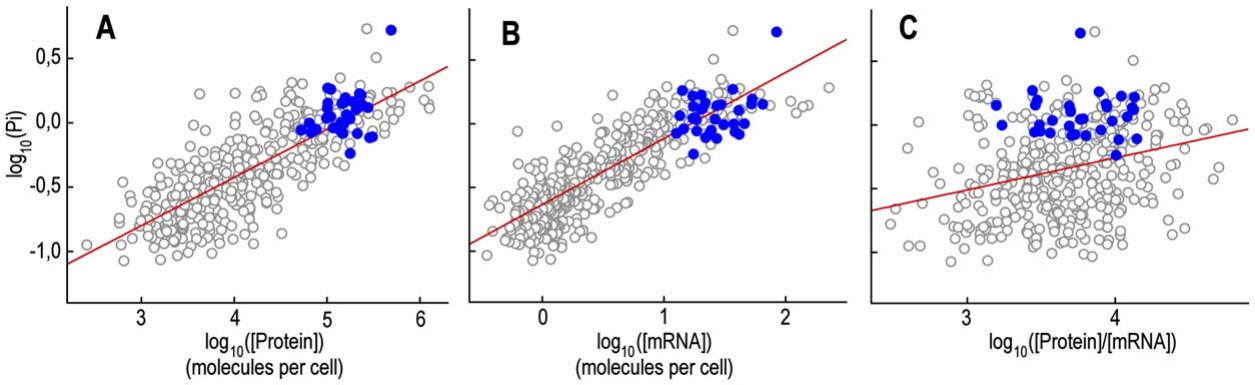
Pi versus gene expression levels in *S. cerevisiae*. Scatter plots of Pi versus cellular protein concentration (panel **A**), cellular mRNA concentration (panel **B**) and protein per mRNA molecule (panel **C**). All values are log-transformed. Blue circles represent genes belonging to the ontology category “structural constituent of the ribosome”.

To calculate the PiCUF, we needed to define a sequence to index and a reference periodicity. The reference periodicity was that expected for a random sequence with a codon composition equal to CUF because we aimed to establish the degree of the relationship between the 3-base periodicity of each CDS and that expected by CUF. However, a periodicity index value for each CDS can be computed by using the expected periodicity resulting from its own codon frequency for both the sequence to index and the reference periodicity. The resulting periodicity index (Pi) is a measurement of the 3-base periodicity expected from the local codon usage. In contrast to CAI, which uses a reference set of highly expressed genes to assess a score [27], Pi is a simple structural parameter that is only related to the codon usage of the gene. As shown above, gene expression levels are strongly correlated only with the codon frequency component of the 3-base periodicity. For these reasons, the correlation studies for examining the relationship between 3-base periodicity and gene expression were performed using Pi. The results show that the Pi of *S. cerevisiae* genes was very strongly correlated with CAI (R_s_ = 0.908 and R^2^ = 0.812), mRNA (R_s_ = 0.854 and R^2^ = 0.719) and protein (R_s_ = 0.803 and R^2^ = 0.642) levels (Table 1 and Figure 7). Similarly to PiCUF, when the mRNA level was partialled out, the strong correlation between Pi and protein level (R^2^ = 0.642) was greatly reduced (partial correlation: R^2^ = 0.082, p<0.0001). Consistently, the correlation between Pi and the protein/mRNA ratio was weak (R_s_ = 0.287 and R^2^ = 0.081).

Transcription of ribosomal protein genes in *S. cerevisiae* is highly coordinated and accounts for 40% of initiation events [28], forming a homogeneous highly-transcribed cluster. Consistent with the above results, the genes of the ontology category “structural constituent of the ribosome” (gene ontology code: GO:0003735) present relatively high values of Pi (blue circles in Figure 7).

In conclusion, the correlation studies of Pi indicate that in yeast, the expected 3-base periodicity resulting from codon composition is strongly correlated with gene expression at the transcriptional level.

### Pi shows a significant positive correlation with transcription rate but not with mRNA stability

**Figure 8:**
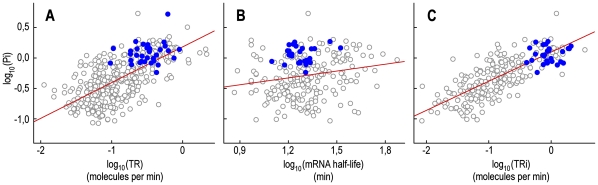
Pi versus rates of transcription and mRNA degradation in *S. cerevisiae*. Scatter plots of Pi versus transcription rate (TR) (panel **A**), Pi versus mRNA half-life (panel **B**) and Pi versus an indirect estimate of the transcription rate (TRi) (panel **C**). All values are log-transformed. Blue circles represent genes belonging to the ontology category “structural constituent of the ribosome”.

We have shown that the strong correlation between Pi and mRNA levels accounts for the majority of the correlation detected between Pi and protein abundance. Because the mRNA abundance (RA) is the result of a kinetic balance between transcription rate (TR) and mRNA degradation rate (DR), we extended the correlation studies by including datasets of transcription rate (TR) and mRNA stability (RS) (usually expressed as half-life) taken from different literature sources [21], [22], [29], [30]. Before presenting the results, it is important to point out some considerations regarding the genome-wide experimental data of TR and RS reported in the literature. In exponentially growing yeast cells, a steady state can be assumed for RA, TR and DR for most of the genes [30]. Under this condition, along with the direct experimental estimation, the TR can be calculated indirectly (TRi) from RA and RS by Formula (1) reported in “Materials and Methods” [21], [30]. Moreover, the genome-wide experimental data for TR reported in the literature are actually a measurement of the density of elongating RNA polymerase II molecules, while the indirect TR, estimated by RS and RA, gives a measurement of the rate related to the mature mRNA [31]. This means that, in contrast to the indirect estimation of TR, the experimental measure of TR does not include the eventual contribution from RNA polymerase speed and pre-mRNA maturation steps, such as splicing and polyadenylation events. This difference between the direct and indirect estimation of TR may not be negligible if we consider, for example, that mRNA elongation speed can vary among different classes of genes [32]. Therefore, any differences found between direct and indirect estimations of TR could be related to the effects of elongation and maturation speed if one of these processes is rate-limiting. Figure 8 shows the scatter plots of Pi versus TR, mRNA half-life and TRi with the corresponding correlation coefficients reported in Table 2. As shown, Pi, CAI and protein levels are strongly correlated with TR and TRi and weakly correlated with the RS values reported by three different authors [21], [22], [29]. This indicates that most of the association of Pi and CAI with transcription takes place at the level of mRNA synthesis rather than mRNA degradation. Moreover, the positive correlations of Pi, CAI and protein level with TRi are higher than those measured with TR, which also suggests a positive correlation with elongation speed and/or maturation rate.

**Table 2:** Correlation coefficients between kinetic data of mRNA and Pi, CAI and protein level of *S. cerevisiae*.

	Pi		CAI^l^		Protein^l^	
	R_s_	R^2^	R_s_	R^2^	R_s_	R^2^
TR^d^	0.665^a^	0.436^a^	0.663^a^	0.453^a^	0.632^a^	0.429^a^
mRNA half-life^e^	−0.212^a^	0.053^a^	−0.257^a^	0.068^a^	−0.259^a^	0.068^a^
mRNA half-life^f^	0.271^a^	0.071^a^	0.283^a^	0.077^a^	0.186^b^	0.042^b^
mRNA half-life^g^	0.167^b^	0.032^b^	0.160^b^	0.037^b^	0.117^c^	0.021^b^
Mean mRNA half-life^h^	0.206^b^	0.039^b^	0.205^b^	0.041^b^	0.128^c^	0.021^b^
TRi^i^	0.793^a^	0.612^a^	0.854^a^	0.725^a^	0.792^a^	0.610^a^

Data are log-transformed.

a *P*<0.0001.

b *P*<0.05.

c *P*>0.05.

d Transcription rate data from the genomic run-on (GRO) technique [30] (N = 389).

e Data from ref. [21] (N = 388).

f Data from ref. [22] (N = 388).

g Data from ref. [29] (N = 245).

h Mean values of data from ref. [21], [22], [29] (N = 234).

i Indirect TR calculated by formula (1) using the mean mRNA half-life (N = 234).

l Data from ref [25].

### Codon biases of alanine, glycine and valine are associated with transcription rate

**Figure 9:**
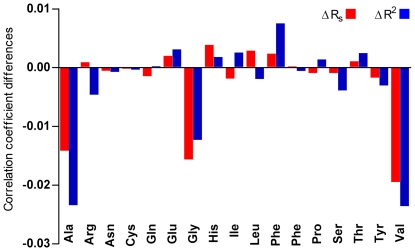
Effects on correlation coefficients between Pi and TR after equalizing synonymous codons of single amino acids in *S. cerevisiae*. Variation of Spearman's rank correlation coefficient (R_s_) (red bars) and the coefficient of determination (R^2^) (blue bars) between Pi (log-transformed) and TR (log-transformed) after equalizing synonymous codons of single amino acids.

**Figure 10:**
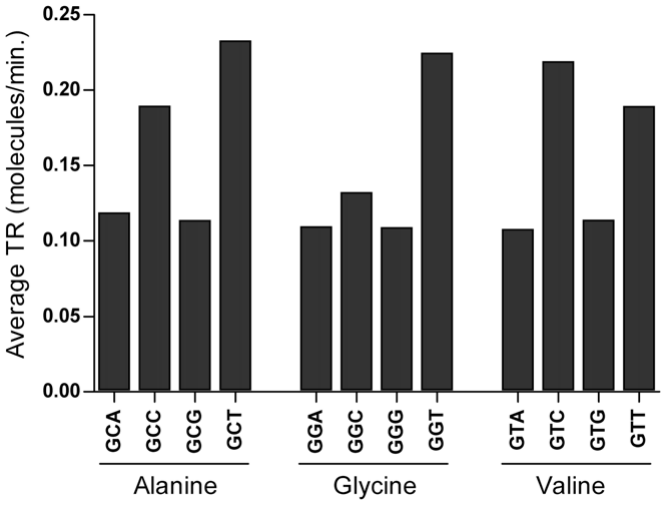
Average TR of codons of alanine, glycine and valine. Average TR of codons belonging to the four-fold degenerate families of alanine, glycine and valine. Each value for the average computing of codon TR was assumed to be equal to the TR measured for the gene where the codon is located.

To evaluate the role of codon bias and amino acid composition in the correlation between TR and Pi, we calculated the correlation coefficients after equalizing the codon frequencies within synonymous sets of codons. The resulting positive coefficients (R_s_ = 0.412, R^2^ = 0.172, p<0.0001, N = 389) show that amino acid composition contributes to the correlation between Pi and TR. To detect which amino acids contribute most to the correlation, we equalized the frequencies of synonymous codons within each amino acid set individually. As shown in Figure 9, the levelling of alanine, glycine and valine codon sets caused the greatest decrease of correlation between the Pi and TR. Alanine, glycine and valine are very small amino acids codified by GC-rich codon families with a four-fold degenerate third codon site: GCA, GCC, GCG and GCT (Ala); GGA, GGC, GGG and GGT (Gly); GTA, GTC, GTG and GTT (Val). The codon bias of these three amino acids is due to the high T content at the third codon position. To establish the distribution of the four synonymous codons among genes with different transcription rates, we computed the average TR for each codon. As shown in Figure 10, C- and T-ending codons of Ala, Gly and Val families are typically associated with genes that exhibit a TR approximately twice that associated with A- and G-ending codons.

### Correlation between Pi and GC content in *S. cerevisiae*

A significant positive correlation was also detected between Pi and the GC content (R_s_ = 0.603 and R^2^ = 0.279, N = 408, p<0.0001) and between GC content and the mRNA level (R_s_ = 0.610 and R^2^ = 0.355, N = 408, p<0.0001) of *S. cerevisiae* genes examined here. From our results, Pi is better correlated with mRNA level than with GC content and is a better predictor of mRNA level than GC content (Table 1). In a previous work it has been shown that, in *S. cerevisiae*, transcripts from GC-rich ORFs accumulate at higher concentrations than those from GC-poor ones [33]. As reported here, G-starting codon families of Ala, Gly and Val are associated with highly transcribed ORFs (Figure 9) and C- and T- ending codons of these amino acids are typically associated with CDSs with high TR (Figure 10). Such a non-uniform distribution of G in the three codon positions may explain part of the correlation between GC content and Pi because the higher the variance of each nucleotide by codon position the higher the periodicity [34]. The replacement of all codons belonging to the Ala, Gly and Val families with the corresponding synonymous G-ending codon results in a overall Pi value lower than those replaced with A-, C- and T-ending ones (data not shown). This shows that, for Ala, Gly and Val , the positive correlation between GC composition and Pi is not valid for the silent third codon position. Thus, although Pi and GC content are both simple measures of codon composition and are independent from codon order, they reflect, at least in part, different properties of codon usage.

## Discussion

The results reported in this study demonstrate, for the first time, that the 3-base periodicity of coding sequences is higher than expected by CUF and is strongly correlated with gene expression at the stage of transcription elongation. We also propose two new periodicity indices: PiCUF, which was employed to investigate the relationship of 3-base periodicity with codon usage, and Pi, which was used to detect the correlation between the codon composition and different levels of gene expression.

The analysis of the 3-base periodicity of coding sequences showed that a codon length of three is a necessary but not sufficient condition to generate 3-base periodicity. The loss of frequency homogeneity, caused by the absence of stop codons inside coding sequences, is sufficient to generate a 3-base periodicity, leading to the consideration that all coding sequences should exhibit this structural property. The removal of codon bias causes strong changes on the expected periodicity of simulated sequences. However, these changes appear to be strongly heterogeneous among different dinucleotides and among sets of simulated sequences based on different CUF. This makes the expected association between the periodicity of individual dinucleotides and codon bias an element of variability among different organisms.

In disagreement with the hypothesis that the periodicity of CDSs could be caused only by CUF [9], [10], in all organisms examined here, which include *M. tuberculosis*, *E. coli*, *B. subtilis* and *S. cerevisiae*, we found that the periodicity of coding sequences is higher than that expected by CUF. We detected two additional sources of 3-base periodicity that were attributed to the position of the amino acids and the synonymous codons within coding sequences. This shows that codons are not randomly positioned within CDSs, suggesting the existence of a force that drives codon sequence in a way that is reflected in the increase of our periodicity index PiCUF.

In *S. cerevisiae*, we also found that the codon frequency and the codon sequence components of PiCUF are differently correlated with gene expression levels. While the codon sequence component of PiCUF does not show any significant association with expression levels, a very strong correlation was detected between the frequency component of the index and the protein as well as the mRNA levels. This difference suggests that, at least in part, the two components of periodicity do not share a common origin and that periodicity itself is not directly associated with gene expression. To limit our study to the analysis of the codon frequency component of the 3-base periodicity, we used the periodicity index Pi. In contrast to PiCUF and CAI, Pi is calculated by using only the codon usage of the coding sequence itself and is a measurement of the amplitude of the 3-base periodicity expected by the codon composition of the gene. The results show that Pi accounted for 71.9% of mRNA variability (R^2^ = 0.719) and 64.2% of protein variability (R^2^ = 0.642) in yeast. Moreover, when the mRNA level was partialled out, the R^2^ between Pi and protein levels (0.642) decreased to a low value (0.082). Thus, this indicates that the mRNA level explains most of the correlation between Pi and protein levels as confirmed by the weak correlation between Pi and the protein/mRNA ratio. This result emphasizes the role of transcription over translation in the relationship between codon composition and gene expression.

It is possible, al least in part, that the correlation between mRNA level and codon usage could reflect translational selection acting to reduce the ribosome bound to highly expressed mRNA and, therefore, increasing free ribosomes and global gene expression [35], [36]. We calculated correlation coefficients between ribosome density for *S. cerevisiae* reported in the work of Arava et al. [37] and Pi index as well as mRNA level of our dataset. Ribosome density showed a significant positive correlation with Pi (R^2^ = 0.42, R_s_ = 0.66, p<0.0001) and with mRNA level (R^2^ = 0.36, R_s_ = 0.65, p<0.0001). The result does not appear consistent with the negative correlation expected if the highly expressed mRNAs sequestered fewer ribosomes. Since in *S. cerevisiae*, density of ribosome decreases with increasing ORF length [37] and is considerably greater for the first 30 to 40 codons [38], we roughly also calculated correlation between Pi and ribosome density by partialling out ORF length obtaining no substantial change of the results.

We performed a supplementary large-scale correlation analysis to evaluate the association of Pi with the activities of the two opposing processes that determine the cellular mRNA level: mRNA transcription and degradation rate [21], [22], [29], [30]. The results showed that the relationship between Pi and mRNA level is only due to the correlation of Pi with the transcription rate. We detected a positive strong correlation between Pi and the density of elongating RNA polymerase II complexes and a very weak correlation of Pi with the mRNA degradation rate. Moreover, in consideration of the arguments about the difference between the direct and indirect measurements of TR discussed in the “Results”, it can be deduced that Pi could also be positively correlated with the polymerase elongation speed and/or maturation rate processes. This is consistent with the finding that the increase in the density of RNA polymerase complexes produces an increase of polymerase speed by apparently preventing backtracking in RNA polymerase complexes in *E. coli* and yeast [39], [40], [41]. We also found that codon bias and amino acid composition both contribute to the correlation between Pi and TR. In particular, pyrimidine-ending codons of the small amino acids alanine, glycine and valine were typically associated with genes with a TR approximately twice that of genes associated with purine-ending codons. In addition, the highly coordinated cluster of genes constituted by the ribosomal proteins, responsible for nearly 40% of the RNA polymerase II transcription initiation events in yeast, exhibited homogeneously high values of Pi.

All of these results indicate that in yeast, the association between codon composition and gene expression levels occurs prevalently at the transcription stage, before translational regulation. This contrasts with the common explanation that attributes the codon bias to translational selection by an adjustment of synonymous codon frequencies to the most abundant isoaccepting tRNA [15], [42]. From our results, it is likely that selection on codon bias acts at both the transcriptional and translational level and that codon usage and the relative abundance of tRNA could drive each other in order to synergistically optimize the efficiency of gene expression. Consistently, expression levels of individual tRNA species in humans are tissue-specific [43], [44], providing evidence of the potential adaptability of tRNA relative abundance to the different demands of gene expression.

It is surprising that the 3-base periodicity of the coding regions, which only depends on codon composition, exhibited a significantly high association with transcription elongation. Correlation does not prove causation; however, considering that 95% of the yeast genes do not contain introns [45], an active role of the coding sequences in maximizing transcription efficiency appears to be the only reasonable explanation for the relationship between the 3-base periodicity and transcription elongation. Consistently, we also found that Pi correlates positively with the GC content of coding regions. In yeast, GC-rich genes tend to be more active and display distinct levels of histone acetylation, which is suggested to be attenuated by the histone deacetylase Rpd3p through a base composition-dependent effect [46]. Our conclusions are also consistent with recent results reporting that coding sequences in yeast could be directly involved in transcription regulation at different post-RNA polymerase II recruitment steps including productive elongation [2], [3]. For example, the RTF1 and SPT5 elongation factors and the CHD1 chromatin remodelling factor associate with coding regions of actively transcribed regions of chromatin, suggesting a regulatory role of chromatin remodelling in transcription elongation [3]. Moreover, an enrichment of RNA polymerase II is detected in intron-less ribosomal protein genes and, at least for RPS3 and RPL25, inactive polymerases accumulate along the length of the gene with some bias toward their 5′ moiety [2]. It seems that codon composition could act by modulating DNA affinity with transcription elongation or chromatin remodelling factors. Another possible mechanism could involve the altered secondary structure of DNA transcribed regions similar to the 3-base periodic secondary structure recently reported for their cognate mRNA coding regions in yeast [47].

In conclusion, this study analysed the 3-base periodicity of coding sequences and its relationship with gene expression by using a new class of periodicity indices. In contrast with earlier studies [9], [10], we show here that the 3-base periodicity of coding sequences cannot be explained by only CUF, but it is also related to the codon sequences. In yeast, we also showed that the 3-base periodicity expected by the codon usage of each individual gene is strongly correlated with the early stage of gene expression at the transcription elongation step. The correlation of codon usage with the transcription level explains most of the correlation observed between codon usage and protein level. These results led to the conclusion that coding sequences should play a key role in maximizing transcription efficiency in exponentially growing yeast cells and that the relative abundances of tRNA isoacceptors may reflect their adaptation to codon usage to maximize gene expression efficiency.

## Materials and Methods

### Genomic sequences and gene expression datasets

Genomic sequence and annotation data for bacterial genomes *Mycobacterium tuberculosis* (NC_000962), *Escherichia coli* (AC_00091) and *Bacillus subtilis* (NC_000964) were downloaded from the NCBI ftp site (ftp://ftp.ncbi.nih.gov/genomes). Coding sequences of *Saccharomyces cerevisiae* were extracted from the non-redundant dataset of CDS (file: cds_nr.fasta) downloaded from the EMBL ftp site (ftp://ftp.ebi.ac.uk/pub/databases/embl/cds) and Saccharomyces Genome Database (SGD) website (http://downloads.yeastgenome.org/sequence/genomic_sequence/orf_dna/).

Unless otherwise specified, to extract and process coding and intergenic sequences, we used software developed in our lab in the C# language. Yeast gene expression datasets including mRNA levels [21], [22], [26], protein abundance [23], [24], [25], codon adaptation index (CAI) [25], transcription rate [30] and mRNA half-life [21], [22], [29] were taken from literature sources. Following the procedure of Lu et al. [25], we used data containing the average concentration for protein and mRNA of at least two of the three reported technologies and genes with a minimum ProteinProphet [48] score *pi* of 0.63 (10% false discovery rate (FDR)) for protein level determined by absolute protein expression (APEX) measurements from mass-spectrometry-based data [25]. This resulted in a subset of CAI, mRNA level and protein level of 408 selected yeast CDSs, which was also used for correlation studies of transcription rate and mRNA half-life. The 408 selected CDSs are 8.3% of the 4924 total verified CDSs in *S. cerevisiae* (*Saccharomyces cerevisiae* database, http://www.yeastgenome.org/cache/genomeSnapshot.html) and represent approximately 30% of the detected cellular mRNA molecules [21], [22], [26]. The data used for the correlation studies are reported in the Dataset S1. Regarding the steady-state condition for mRNA abundance (RA) in exponentially growing yeast [30], the indirect transcription rate (TRi) was estimated by an experimental measurement of mRNA abundance (RA) and mRNA stability (RS) by the following formula [21], [30]:
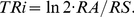
(1)


### D_XY_ frequency distributions

DNA periodicity in coding regions was studied by analysing the frequency distribution of the discrete variable D_XY_ that measures the waiting time of the first occurrence of nucleotide Y after nucleotide X. The D_XY_ frequency distributions of all of the 16 possible X-Y couples of nucleotides were calculated for each real or simulated CDS. For graphical illustration, D_XY_ frequency distributions were represented in units of expected frequency for the RandNuc model, defined as the frequency equivalent RandNuc (FeRandNuc). Before computing D_XY_, the first and last codons of the CDSs were removed.

### Simulated model sequences

Three principal classes of random sequence models were generated:

- RandNuc: random nucleotide sequences generated using a 0-order Markov model with a specified probability for each nucleotide.
- RandCod: random codon sequences generated using a 0-order Markov model with a specified probability for each codon.
- ShufCod: random sequences produced by randomly shuffling codons within each coding sequence deprived of its first and last nucleotide triplets.

RandNuc and RandCod sequences were generated by the software GenRGenS [49]. ShufCod sequences were generated using the Fisher-Yates algorithm [50]. For simulation procedures, nucleotide and codon frequencies from native CDSs were computed without considering their first and last codons. As an example, each simulation related to the coding sequences of *M. tuberculosis* produced 5000 RandNuc or RandCod sequences of 2866752 nucleotides, which is the total length of CDSs used in this work. Before calculating D_XY_ frequency distributions, each of the 5000 simulated sequences was fragmented into 2388 parts to reproduce the number and length of real CDSs. In total, 5000 sets of ShufCod sequences were generated by 5000 random permutations of codons within each coding sequence.

### Theoretical distributions

In addition to the estimated values from simulated sequences, the expected values for the D_XY_ frequency distributions of RandNuc and RandCod were calculated. In the case of RandNuc sequences, the D_XY_ discrete variable presents a geometric distribution with the following probability mass function: 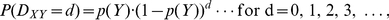
(2)where p(Y) is the probability of occurrence of nucleotide Y estimated from its relative frequency in real coding sequences.

For the RandCod model, the probability mass function is the following:
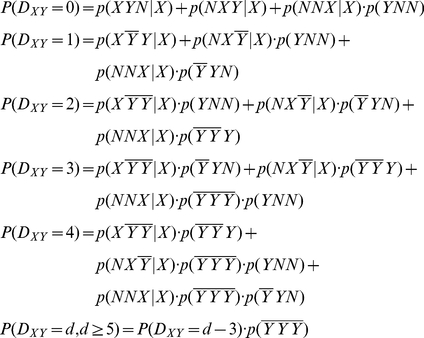
(3)where 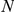 indicates any nucleotide, and 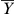 indicates any nucleotide but 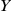. Probabilities were estimated from the codon and nucleotide frequencies of the real coding sequences. For example, P(ATN|A) was estimated by the absolute frequency of the ATN codons divided by the absolute frequency of A in the real coding sequences. In the case of a finite sequence of length 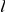, the expected number of times 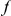 that nucleotide Y occurs after 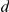 nucleotides from X is equal to the following:
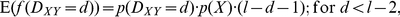
(4)where 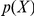 is the probability of occurrence of nucleotide X estimated from its relative frequency in real coding sequences.

### Periodicity index calculation

The periodicity index PiCUF depends on the sum, over all 16 dinucleotides, of the difference between each couple of contiguous D_XY_ frequency points multiplied by the corresponding expected differences for RandCod sequences based on codon usage frequency (CUF). In this work, CUF was intended as the global codon composition of the analysed real CDSs lacking the first and last codons.

PiCUF was computed by the following formula: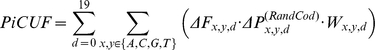 Where:
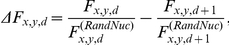


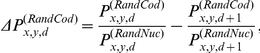


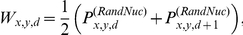




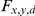
 is the frequency of the first occurrence of base *Y* after *d* nucleotides from base *X* measured in the sequence to score,


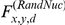
 is the calculated frequency for the RandNuc model using the nucleotide composition of sequence to score,


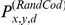
 is the probability for the RandCod model at codon composition equal to CUF,


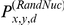
 is the probability for the RandNuc model at nucleotide composition of sequence to score.

The Pi index was computed similarly to PiCUF but by using the RandCod sequence based on the codon frequencies of the sequence to score for the sequence to index and the reference sequence. D_XY_ and CUF were computed after removing the first and last codons of CDSs. Software written in C# was used to compute the D_XY_ frequency distributions of native and simulated sequences and their periodicity indices (manuscript in preparation). All software was tested by independent computational tools and manual calculations.

### Statistical analysis of correlation studies

The statistical analysis was performed using standard parametric and non-parametric tests included in the Statistica package (version 8.0, Statsoft, Inc.). The statistical significance of the difference between the PiCUF mean value of native and shuffled CDSs was evaluated by simulating the distribution of the mean of 5000 sets of shuffled CDS. The normality of the distribution was tested using the Shapiro-Wilk normality test and normal probability plot.

## Supporting Information

Figure S1 **D_TT_ frequency distributions of RandCod based on equal codon frequencies.** Comparison between the D_TT_ frequency distributions of RandCod artificial sequences based on equal frequencies for all codons with (blue points) or without stop codons (red points).
(TIF)

Figure S2 **Best sine-wave fit of D_TT_ frequency distribution of RandCod and CDS.** D_TT_ frequency distribution of RandCod (panel **A**) and CDS (panel **B**) sequences with the best sine-wave fit (red line).
(TIF)

Figure S3 **PiCUF in CDSs of S. cerevisiae and artificial sequences.** Frequency distribution diagrams of PiCUF in CDSs of *S. cerevisiae* (a) and artificial sequences: *S. cerevisiae* CDSs with synonymous codons shuffled (b), *S. cerevisiae* CDSs with codons shuffled (ShufCod) (c), RandNuc based on *S. cerevisiae* nucleotide frequencies (d). Absolute frequencies of real CDSs were normalized against frequencies of simulated sequences by multiplying by 5000.
(TIF)

Figure S4 **PiCUF versus CAI and expression levels.** Scatter plots of PiCUF and its sequence and frequency components versus log-transformed CAI, cellular protein level, cellular mRNA level and protein/mRNA ratio.
(TIF)

Table S1 **Mean PiCUF values of CDSs of M. tuberculosis, E. coli, B. subtilis and S. cerevisiae.**
(DOC)

Dataset S1 **Data used for the correlation studies between the periodicity indices and gene expression levels.**
(XLS)
