# Supplementary figures and images for: The 3-Base Periodicity and Codon Usage of Coding Sequences Are Correlated with Gene Expression at the Level of Transcription Elongation
Source: PLoS One. 2011 Jun 28;6(6):e21590. doi: 10.1371/journal.pone.0021590 (PMC3125259; doi:10.1371/journal.pone.0021590)

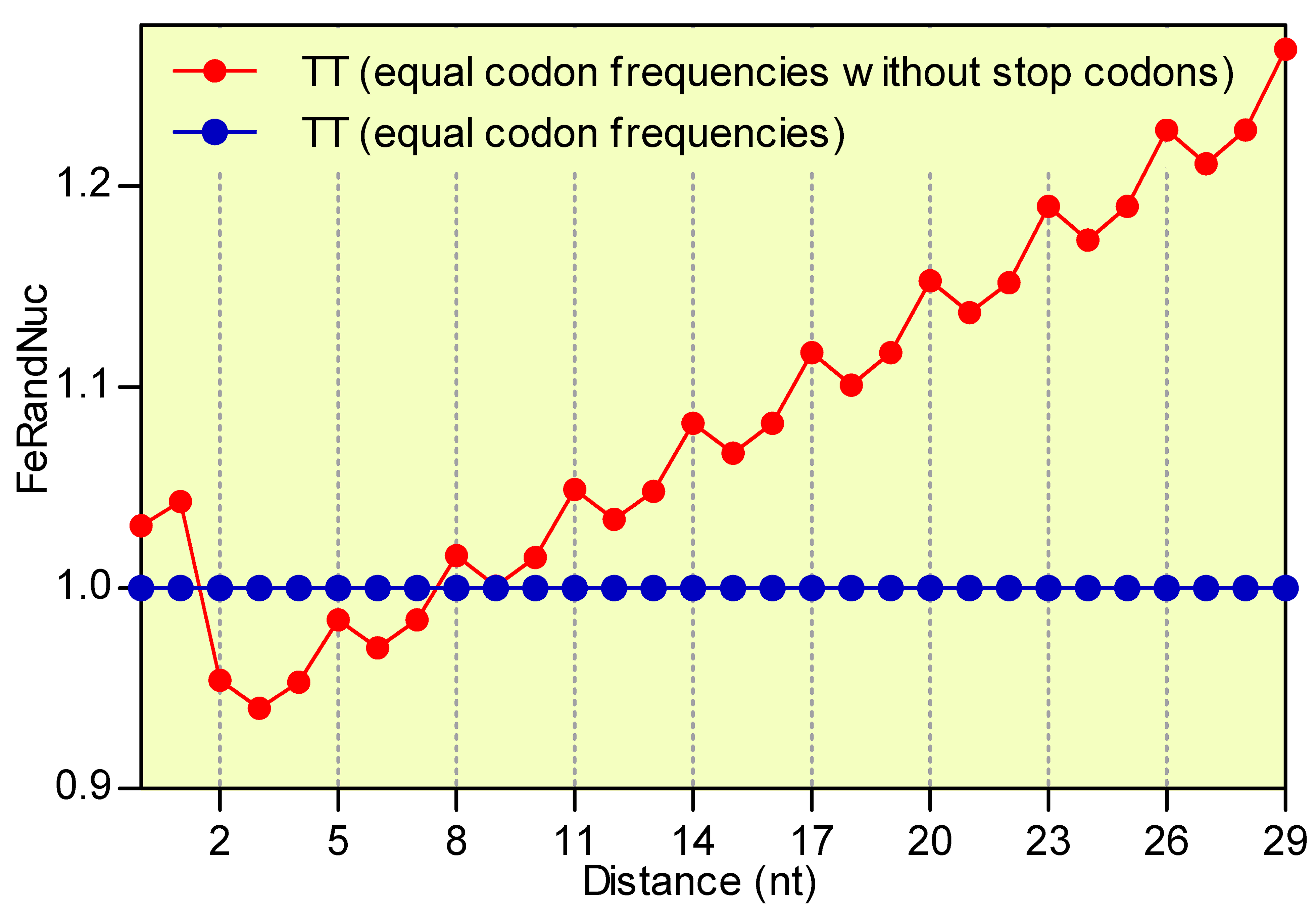

Supplement: Figure S1 — DTT frequency distributions of RandCod based on equal codon frequencies. Comparison between the DTT frequency distributions of RandCod artificial sequences based on equal frequencies for all codons with (blue points) or without stop codons (red points). (TIF) [file pone.0021590.s001.tif]

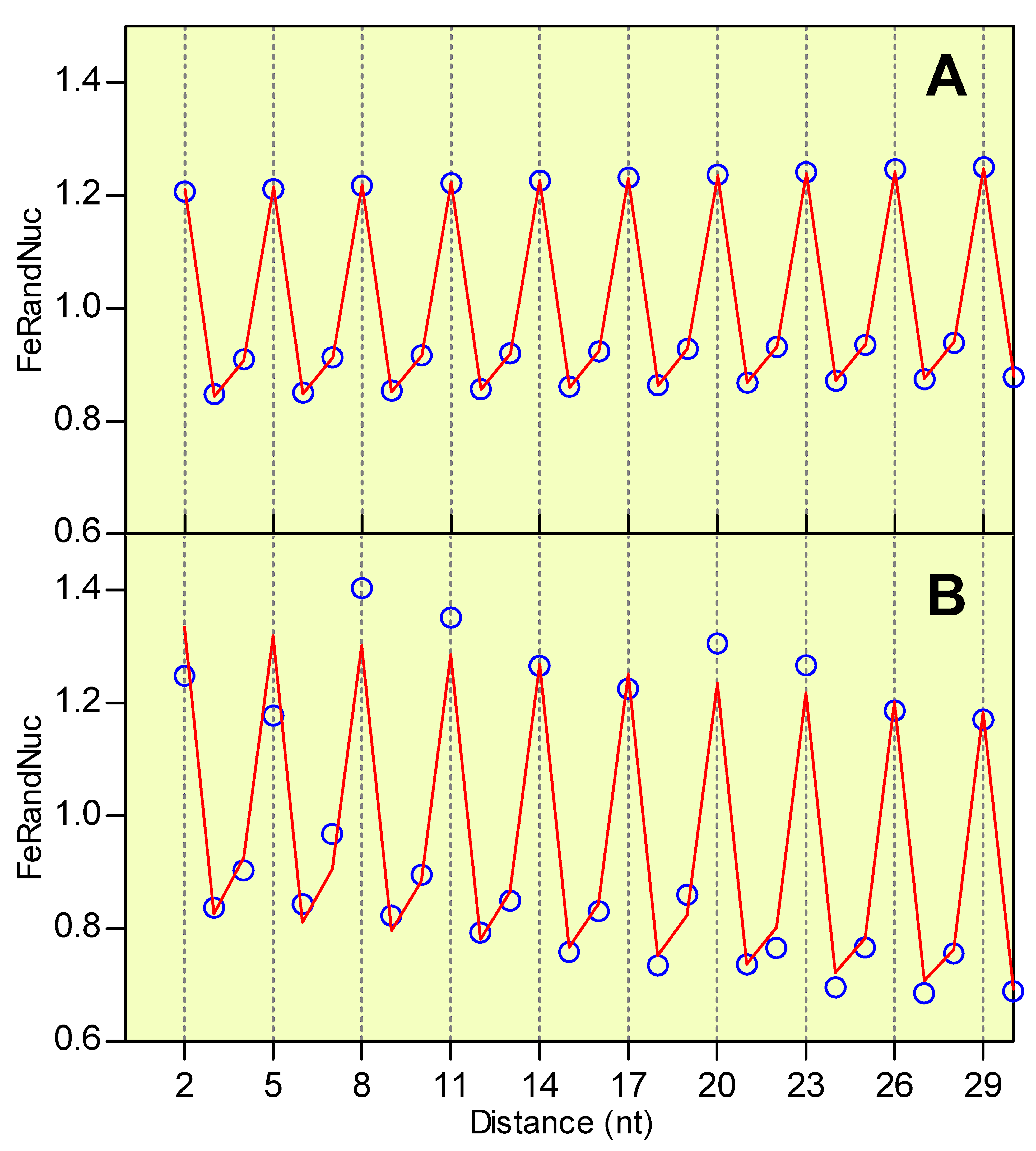

Supplement: Figure S2 — Best sine-wave fit of DTT frequency distribution of RandCod and CDS. DTT frequency distribution of RandCod (panel A) and CDS (panel B) sequences with the best sine-wave fit (red line). (TIF) [file pone.0021590.s002.tif]

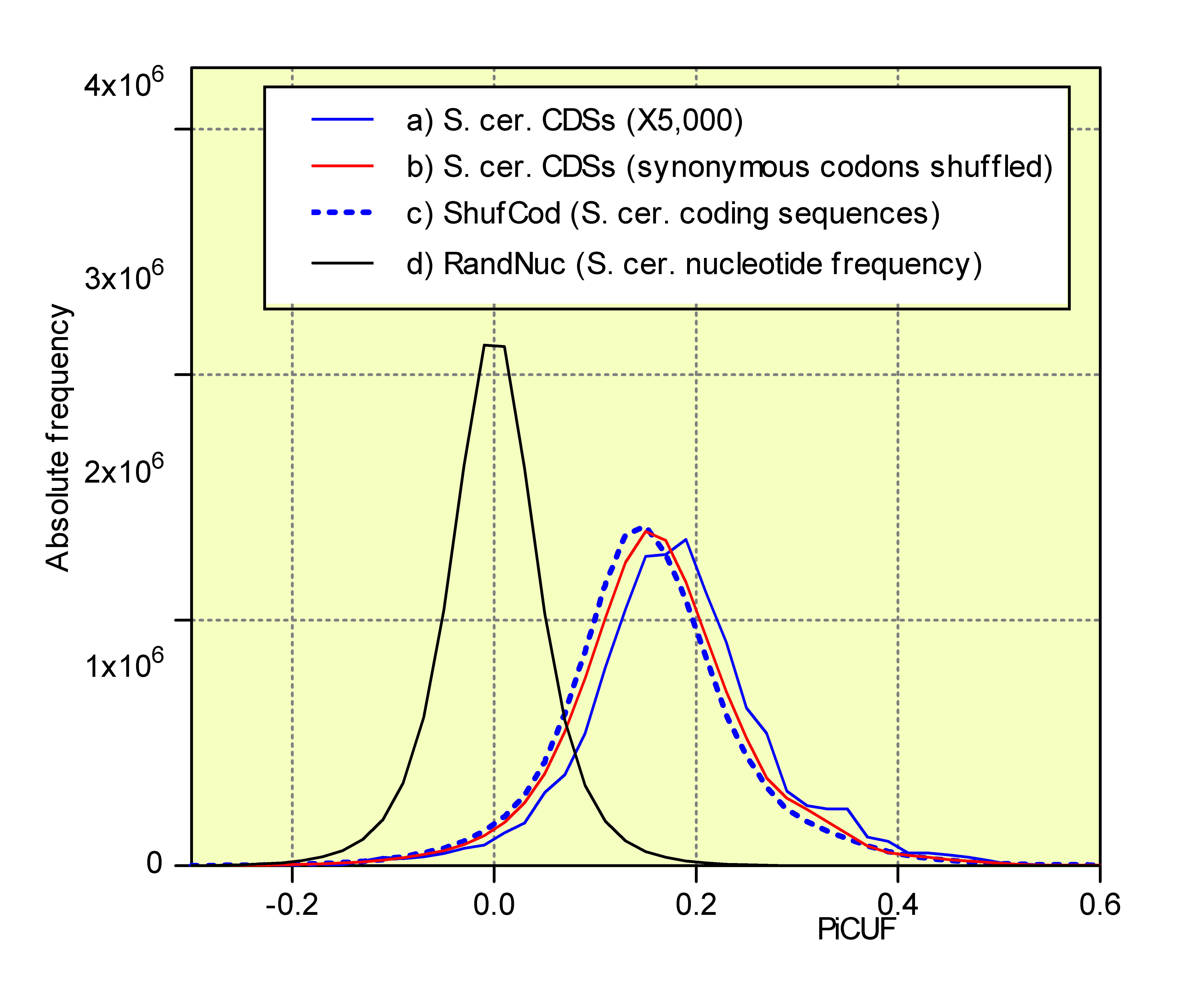

Supplement: Figure S3 — PiCUF in CDSs of S. cerevisiae and artificial sequences. Frequency distribution diagrams of PiCUF in CDSs of S. cerevisiae (a) and artificial sequences: S. cerevisiae CDSs with synonymous codons shuffled (b), S. cerevisiae CDSs with codons shuffled (ShufCod) (c), RandNuc based on S. cerevisiae nucleotide frequencies (d). Absolute frequencies of real CDSs were normalized against frequencies of simulated sequences by multiplying by 5000. (TIF) [file pone.0021590.s003.tif]

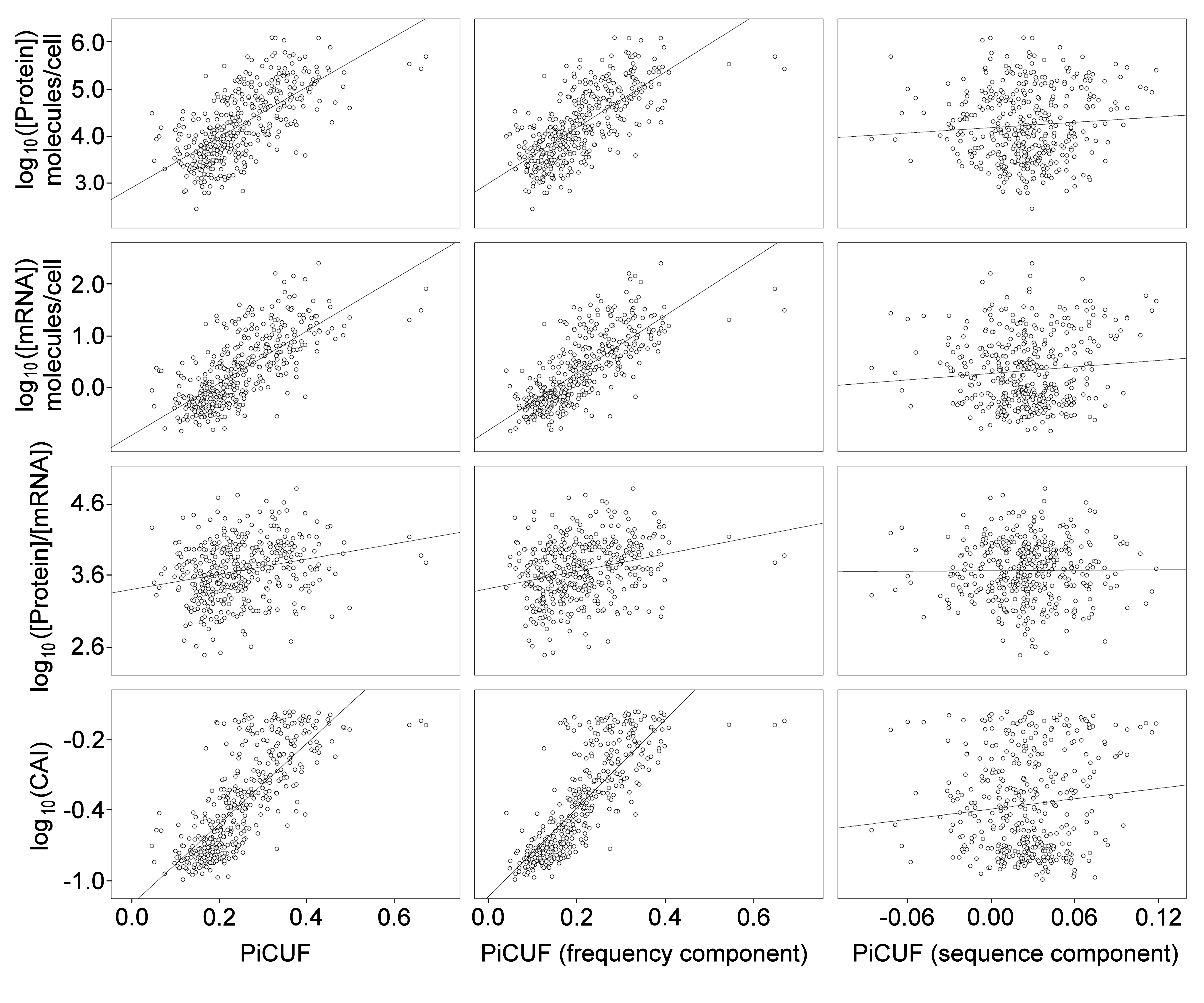

Supplement: Figure S4 — PiCUF versus CAI and expression levels. Scatter plots of PiCUF and its sequence and frequency components versus log-transformed CAI, cellular protein level, cellular mRNA level and protein/mRNA ratio. (TIF) [file pone.0021590.s004.tif]
