# Supplementary material for: The 3-Base Periodicity and Codon Usage of Coding Sequences Are Correlated with Gene Expression at the Level of Transcription Elongation
Source: PLoS One. 2011 Jun 28;6(6):e21590. doi: 10.1371/journal.pone.0021590 (PMC3125259; doi:10.1371/journal.pone.0021590)
Supplement: Table S1 — Mean PiCUF values of CDSs of M. tuberculosis, E. coli, B. subtilis and S. cerevisiae. (DOC) [file pone.0021590.s005.doc]

| **Table S1 .**  Mean PiCUF values of CDSs of M. tuberculosis, E. coli, B. subtilis and S. cerevisiae.. | | | |
| --- | --- | --- | --- |
| Organism | CDSs | | |
|  | Native | All codons shuffled | Synonymous codons shuffled |
| M. tuberculosis | 0.527 (100%) | 0.436 (82.7%) | 0.463 (87.9%) |
| E. coli | 0.394 (100%) | 0.323 (82.0%) | 0.335 (85.0%) |
| B. subtilis | 0.318 (100%) | 0.225 (70.8%) | 0.243 (76.4%) |
| S. cerevisiae | 0.182 (100%) | 0.155 (85.2%) | 0.164 (90.1%) |
